# Mapping the Second Victim Experience Among Western Nurses: A Scoping Review

**DOI:** 10.3390/healthcare14040467

**Published:** 2026-02-12

**Authors:** Cristina Costeira, Helena Junqueira, Pedro Quintas, Ângela Pragosa, Ema Mata, Hugo Duarte, Luís Bom, Nelson Pais

**Affiliations:** 1 School of Health Sciences of Polytechnic University of Leiria (ESSLei), Campus 2, Morro do Lena, Alto do Vieiro, Apartado 4137, 2411-901 Leiria, Portugal; angela.pragosa@ipleiria.pt (Â.P.); ema.mata@ipleiria.pt (E.M.); hugo.duarte@ipleiria.pt (H.D.); luis.bom@ipleiria.pt (L.B.); 2 Center for Innovative Care and Health Technology (ciTechCare), Polytechnic University of Leiria, Campus 5, Rua das Olhalvas, 2414-016 Leiria, Portugal; 3 Health Sciences Research Unit: Nursing (UICISA: E), Nursing School of Coimbra University (ESEUC), 3004-011 Coimbra, Portugal; 4 Medical Service of Pombal Hospital, Local Health Unit of the Leiria Region (ULSRL), Hospital de Pombal Pombal, 3100-462 Leiria, Portugal; helena.junqueira@ulsrl.min-saude.pt; 5 Pombal Community Care Unit, Local Health Unit of the Leiria Region (ULSRL), Avenida Heróis do Ultramar 90 Pombal, 3100-462 Leiria, Portugal; pedro.quintas@ulsrl.min-saude.pt; 6 Pain Unit, Portuguese Oncology Institute of Coimbra (IPOC), 3004-011 Coimbra, Portugal; 1486@ipocoimbra.min-saude.pt

**Keywords:** nurse, medical errors, occupational stress, adverse effects, psychological trauma, patient safety, second victim phenomenon, second victim syndrome

## Abstract

Background/Objectives: The second victim phenomenon is increasingly recognized as a significant issue affecting nurses involved in adverse events resulting from clinical decisions or interventions. Although patients and families, considered the first victims, are directly impacted, nurses often undergo challenges as second victims. With the growing awareness of these effects, this study aimed to map recent evidence on the second victim phenomenon among nurses in Western countries. Methodology: A Scoping Review was conducted following the Joanna Briggs Institute methodology in September 2024 and updated in November 2025. Eligibility criteria were defined using the PCC (Population, Concept, Context) framework. Searches were performed in PubMed, CINAHL, SciELO, and Scopus. Two independent reviewers carried out study selection, data extraction, and synthesis. Rayyan^®^ supported screening, performed in two phases: title/abstract review and full-text analysis. Data extraction was conducted in Excel^®^, and data were analyzed using descriptive statistics and categorized into thematic areas. The review followed PRISMA-ScR guidelines and was registered in the Open Science Framework. Results: Of the 111 articles retrieved, 39 met the inclusion criteria. Evidence shows that although several support programs exist for nurses as second victims, they are often perceived as inadequate or inconsistently implemented. Second victim experience is associated with physical (e.g., sleep disturbances), emotional (e.g., fear), and psychological (e.g., distress) symptoms, with consequences such as absenteeism, professional dissatisfaction, loss of meaning in life, and even suicide. Conclusion: Findings highlight the need for more comprehensive, accessible, and consistently implemented support strategies to meet the complex needs of nurses affected by the second victim phenomenon.

## 1. Introduction

Medical errors represent a major public health concern in the United States and are ranked as the third-leading cause of death after heart disease and cancer [1,2].

The Institute of Medicine report “To Err Is Human” emphasizes the critical importance of preventing such errors, especially in healthcare, where the consequences for first victims (patients and their families) can be devastating and irreversible, including death and permanent disability, and where errors can have a significant impact in healthcare professionals [3,4]. When errors occur, a domino effect ensues, affecting four groups: the patient and family (first victims), healthcare professionals (second victims), the institution’s reputation (third victim), and patients who are subsequently harmed (fourth victims) [1].

In this context, the concept of the second victim phenomenon emerged, as originated by Albert Wu [5], to characterize the emotional and psychological impact of medical errors on healthcare professionals [5].

The second victim phenomenon describes the impact of errors causing preventable patient harm on healthcare professionals [6,7]. It is also referred to as second victim syndrome, which describes the significant emotional, psychosomatic, and psychological distress experienced by healthcare professionals following adverse patient events [8,9]. In this paper, we adopt the broader concept of the second victim phenomenon to frame our analysis, clearly distinguishing it from the more clinical framing of second victim syndrome.

Given nurses’ central role in direct patient care, care coordination, medication administration, and clinical surveillance, nursing practice is closely linked to both the prevention and occurrence of medical errors. Nurses are the health professionals most affected by the second victim phenomenon [10]. Representing the largest professional group within the healthcare workforce, they are exposed to continuous pressures and complex challenges, such as heavy workloads, frequent rapid response interventions, and the management of critically ill patients, which increase their susceptibility to errors [11]. According to Strametz et al. [12], 59% of nurses experience the second victim phenomenon at least once during their careers.

It is known that unmitigated recovery of a second victim can contribute to job insecurity, absenteeism, turnover intentions, increase invasive rumination, and a loss of joy and meaning in work [13,14,15,16]. Other researchers identify additional consequences from this phenomenon in daily nursing life, such as loss of self-confidence and self-efficacy, shame, self-blame, doubts, insomnia, stress, low self-esteem, guilt, helplessness, inability to think, confusion, exhaustion, eating difficulties, sickness, and depression [7,17].

Recognizing the second victim phenomenon as an important occupational concern, some institutions have developed employee assistance programs and implemented support services, including chaplaincy, counseling, and stress management resources [18]. Despite these efforts, many healthcare organizations continue to offer limited support for their nursing professionals [19].

The literature also reports that the second victim phenomenon is still neglected in medical and nursing curricula in European universities [20].

This phenomenon has generated, in the last years, a special interest among researchers [10]. However, concerns remain regarding the institutional support desired by nurses, consequences for their personal and professional life, and the profile of the nurses who suffer more with this phenomenon in Western countries. The term “Western countries” refers to nations primarily situated in Europe, North America, and Oceania, which share broadly comparable healthcare systems, professional nursing structures, and patient safety cultures [21,22]. In this study, we also included countries such as Brazil and Israel based on these criteria, considering similarities in healthcare organization and nursing practice, even if they are not intuitively classified as “Western” by all readers. This explicit definition clarifies our inclusion criteria and ensures transparency regarding the selected countries.

Consequently, this study was designed to map recent evidence on the second victim phenomenon among nurses in Western countries, with the goal of informing nurse managers and raising their awareness of the consequences and challenges that nurses, as second victims, are required to navigate.

## 2. Materials and Methods

A literature review was conducted in September 2024 and updated in November 2025, following the methodological guidelines of the Joanna Briggs Institute (JBI) for Scoping Reviews (SRs) [23].

The pre-established five steps were (i) formulation of the research question; (ii) identification of the relevant sources of evidence; (iii) the selection of sources of evidence for inclusion; (iv) data collection/extraction; and (v) grouping, summarizing, and reporting results [18].

The authors developed a search strategy and protocol. The research team comprised nursing academy members (C.C., Â.P., L.B., E.M., H.D.) and clinical nurses (H.J., P.Q., N.P.). Three of the authors have training and experience in JBI methodology to conduct SRs.

During the literature search, a systematic review by Naya et al. [9] on intensive care, as well as reviews by Sahay et al. [7] and Catalán et al. [10], were identified as addressing the concept. However, no review specifically focused on the research questions identified in the present study, justifying the development of this review.

The review is reported in accordance with the PRISMA extension for SR (PRISMA-ScR) [24] (Supplementary File S1). The protocol was registered a priori in the Open Science Framework (https://doi.org/10.17605/OSF.IO/AGMZ2).

The PCC mnemonic (Population, Concept, and Context) was used to structure the research question and establish eligibility criteria [23,25].

In accordance with the JBI methodology for scoping reviews, a critical appraisal of the included studies was not conducted, as the primary aim of this review was to map and describe the available evidence rather than assess study quality.

### 2.1. Research Question

This study addresses the following research questions:

1. What are the specific characteristics of the healthcare services or institutions where nurses are most frequently reported as second victims?
2. What types of support do nurses receive or have access to as second victims?
3. What are the most reported symptoms experienced by nurses identified as second victims?
4. What types of incidents or errors are most frequently reported as leading nurses to become second victims?
5. What forms of support are preferred and desired by nurses who experience the second victim phenomenon?
6. What are the most frequently reported consequences for nurses affected by the second victim phenomenon?

### 2.2. Eligibility Criteria

No linguistic filters were applied as exclusion criteria for this review. The year 2000 was established as the starting point, as the concept first appeared in the literature at that time [4]. All types of documents referring to nurses and the second victim phenomenon or second victim syndrome in Western contexts were included. Table 1 presents the PCC framework used to define the eligibility criteria.

### 2.3. Search Strategy

The search strategy, carried out in three stages, as established by the JBI framework for scoping literature reviews [24], sought to locate primary studies, literature reviews, and technical documents published until September 2024 and updated on 15 November 2025, to obtain current and relevant scientific evidence and technical documents directly related to the topic under study. At the first stage, on 5 September 2024, a limited search was conducted on Medline via PubMed, using truncation and operators (Boolean OR and AND). Table 2 shows the search strategy in the MEDLINE database. At this stage, subject headings and keywords in titles and abstracts were analyzed to inform the planning of a subsequent search.

In the second phase (20 September 2024 and an update on 15 November 2025), a search was conducted in four databases [MEDLINE via EBSCO; Scopus; CINAHL via EBSCO; Scientific Electronic Library Online (SciELO)]. The search strategies were adapted to each database. Finally, in the third phase, the reference lists of the included studies were transferred to the Rayann software^®^ (Qatar Computing Research Institute, Doha, Qatar).

### 2.4. Study Selection

The selection of documents followed a rigorous methodology structured in two main stages: screening of titles and abstracts and full-text reading, always carried out by two independent researchers (using a blind-on process). Initially, n = 111 were identified and were exported and uploaded to Rayyan^®^ software (Qatar Computing Research Institute, Doha, Qatar) for the initial elimination of duplicates. At the second stage, the studies eligible for review were passed on for full-text reading (n = 61) and again checked against the established eligibility criteria, which was also done using two independent, blinded reviewers. Reasons for exclusion were standardized and reported in the two stages of screening. Conflicts between reviewers (C.C. and H.J.) were resolved with the intervention of a third reviewer (P.Q.). It was previously established that, if any information was missing, the corresponding author would be contacted whenever the data available in the paper were insufficient or unclear. If the corresponding author did not respond within four weeks, after two different email attempts (every two weeks), and the missing information was essential to ensure the reliability of the extracted data, the study was excluded.

**Figure 1:**
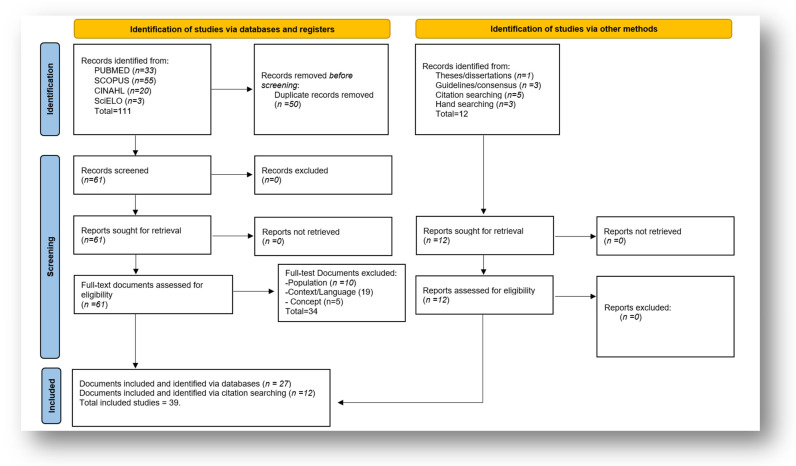
PRISMA-ScR flowchart for identifying, screening, and selecting the articles included in the Scoping Review.

One article had missing information; the necessary clarifications were requested and successfully obtained. The PRISMA flowchart (Figure 1) presents the total number of records identified, as well as the reports included and excluded, indicating the reasons for exclusion and the documents added after manually checking the reference lists.

### 2.5. Data Extraction

Data were extracted and synthesized by two authors independently in pairs (C.C., H.J., P.Q., N.P.). Any disagreements between authors were discussed/analyzed with a third reviewer (P.Q. or C.C. depending on the pair of researchers).

The extracted data were based on an instrument developed for this purpose by the authors in Microsoft Excel^®^.

For each study included, the following data were extracted: title; authors; year of publication; country; setting (service, unit, or institution); objectives; study type; sample and data collection instrument characteristics; main findings; and specific information: types of support provided; prevalent symptoms among nurses as second victims; most frequent incidents reported and affecting first victims; preferred support for second victims; and consequences that nurses experience after a clinical error.

### 2.6. Data Synthesis and Reporting

The main findings were aggregated, summarized, and presented in a single document and were organized according to the key themes, including workplace characteristics where nurses developed them actively during the event; types of support provided; prevalent symptoms among nurses as second victims; the most frequent incidents reported affecting first victims; preferred type of support for second victims; and consequences to second victim nurses. The characteristics of the studies were summarized in tables using descriptive statistics via Microsoft Excel^®^ and narrative description from thematic categorization. Concept-related data were organized and categorized into key themes by content analysis.

## 3. Results

The search, conducted between 5 September 2024 and 15 November 2025, identified 111 documents in the databases, including those added during the update, which contributed six additional documents [11,26,27,28,29,30]. Duplicates (n = 50) and documents that did not address the research questions or did not meet the inclusion criteria were excluded (n = 34). Among these, an additional 12 documents were included through searches of the bibliographies of the retrieved articles and relevant technical documents related to the topic.

In the end, a total of 39 articles were included in the SR (Supplementary File S2). The results of the search are shown in a flow diagram in Figure 1.

All retrieved documents were published between 2015 and 2025. It is important to highlight that the majority of the retrieved documents were published after 2019, in the post-COVID period (84.6% of the documents included in the review).

Most were primary studies (n = 19) [ i.e., qualitative studies; cross-sectional studies], with eight secondary studies [i.e., scoping reviews; systematic reviews; literature reviews] and twelve categorized as other designs [policy briefs (n = 1); master thesis (n = 1); guidelines (n = 3); technical recommendations (n = 3); methodological studies (n = 1); quality improvement projects (n = 2); legal documents (n = 1)].

The publication language was English in 34 documents and Portuguese in five.

**Figure 2:**
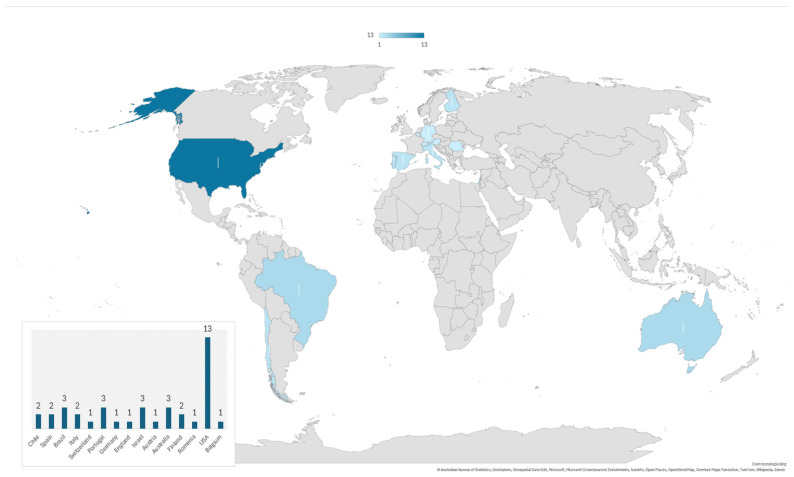
Geographic distribution of studies included in the scoping review.

The country that contributed the largest number of documents was the United States (33%) (n = 13), followed by Portugal (n = 3), Israel (n = 3), Brazil (n = 3) and Australia (n = 3) (Figure 2).

In response to the question, *“1—What are the specific characteristics of the healthcare services or institutions where nurses are most frequently reported as second victims?”*, the extracted data indicate a wide variety of care settings and nursing backgrounds. Reports include nurses working in inpatient units [31,32], pediatric services [26,33], intensive care units [34,35,36], emergency departments [11], obstetrics and gynecology services [37], and operating rooms [4,14,38], as well as newly graduated nurses [17]. However, most documents referred broadly to nurses, either direct care providers or nurse managers, without specifying the clinical area or focused on nurses identified as second victims or participating in support programs [12,19,29,39,40,41,42,43,44,45,46,47,48,49,50,51,52,53,54,55,56,57].

The documents analyzed in response to the second question, “*2—What types of support do nurses receive or have access to as second victims?*”, allowed the categorization of support into two main categories, **institutional support and personal support**, each of which can be further divided into **formal and informal strategies**.

The documents reviewed indicate the existence of **formal** programs, protocols, and resources for emotional support as offered by institutions [4,11,12,34,43,47]. However, not all nurses identified as second victims were aware of these institutional initiatives [17], and many classified the institutional response as insufficient or lacking [19,27,32,40].

The **formal strategies** identified include access to counselling and psychological/psychiatric services; opportunities to discuss emotional and ethical issues with structured peer groups and managers; clear information regarding institutional processes (e.g., root-cause analysis, incident reporting); prompt debriefing or crisis intervention; supportive guidance for continuing clinical duties; assistance with communicating with patients; clarification of roles and expectations after the incident; support in actively participating in post-incident review processes; safe opportunities to contribute insights to prevent similar events; legal guidance after the incident; and the possibility of taking immediate time off to recover [12,44]. Nonetheless, the literature shows that the support nurses rely on is predominantly informal and often provided outside the institution [17,32,33,34].

Some nurses reported that institutions provide educational and training programs aimed at improving clinical practices, promoting patient-centered care, and addressing adverse events. However, specific policies or structured programs dedicated to supporting second victims remain limited [7,35,38].

The most frequently identified **informal strategy** across the documents is peer support, followed closely by support from family and friends, which represent the primary forms of personal support for nurses who become second victims [12]. Finney et al. [37] further reports that peer support is provided in 95.5% of cases, while support from family members occurs in 63.6% of cases, highlighting its significance as a key source of assistance.

In the third question of this review, “*3—What are the most reported symptoms experienced by nurses identified as second victims*?”, a wide diversity of symptoms is described. These symptoms can be categorized into **physical, emotional, and psychological symptoms.**

**Physical symptoms** reported by nurses include lethargy [12], sleeping difficulties [33,34,48], insomnia [4,29,34], nausea [4], headaches [19], sweating [19,34], racing heart [19,34], hot flashes [19], dizziness [19], palpitations [34], tiredness [29] and exhaustion [7].

**Emotional symptoms** identified include guilt [12,38], shame [38], self-blame [12,35,38], and anger toward oneself and others [12].

The most frequently reported emotional reaction is fear, which may take several forms: fear of losing one’s job [12], fear of judgment, institutional disrespect, reprimands, contempt, retaliation, punishment, colleagues’ reactions, potential consequences, disciplinary actions, lawsuits, fear for the patient’s well-being and of the patient’s reactions [30,46], and fear of not being competent or capable of providing care again [38].

In the **psychological dimension**, the most frequently reported symptoms include intrusive memories [28,38], distress [7,14,28,33,35,43] and anxiety [4,7,12,19,29,33,35,38,46].

Other psychological symptoms include self-stigmatization [34], depression [4,7,12,19,28,33,34,35,38,46], denial [46], distortion of reality [46], post-traumatic stress disorder [11,46], mental anguish [19], aggressive behaviors [34], low self-esteem [7], inability to think clearly [7], confusion [7], and a sense of burden [27].

Only a few documents identify the “4*—(…) types of incidents or errors most frequently reported as leading nurses to become second victims*.” Those incidents or errors can be categorized by their **consequences for the victims** (minor or severe errors) and by the **phase of care in which they occur** (diagnostic, treatment, or prevention).

The most severe incidents reported were death or suicide of a patient or colleague [4,12,38], fetal or neonatal loss, maternal death, intrapartum or neonatal death, and patient death resulting from lack of continuity of care, across all phases of treatment [37].

Other clinical errors identified include medication errors, whether in prescribing, preparing, or administering medications [17,38]. Missed diagnoses [37] and errors related to medical procedures [12,46] were also identified as frequent incidents or errors, though they were often considered minor when they did not result in patient death. It is widely agreed that implementing multiple preventive interventions is essential to ensure the safety and quality of healthcare delivery, thereby minimizing incidents/errors and preventing negative consequences for all stakeholders [51,52,53,55,57].

Nurses reported the “*5—(…) preferred and desired forms of support for those experiencing the second victim phenomenon*” as falling into two main categories: **institutional support** and **support outside the institution**.

**Within the institution**, nurses emphasized the importance of being able to take a day off after the adverse event [35]. Many highlighted the value of receiving support from colleagues and supervisors [4,7,19,35,36], as well as having opportunities to share their experiences with peers [4,19,35]. Nurses also expressed a desire for structured support programs [28,33,37,53,54] and access to psychological counselling and emotional support [7,28,30]. They further underscored the importance of discussing events openly and without judgment [32], as well as being involved in the review process to offer insights on how similar incidents could be prevented in the future [46].

**Outside the institution**, nurses stated that they would appreciate support from family and friends [18]. They also expressed the wish for policymakers to prioritize the implementation of standardized peer-support frameworks and resilience-training programs within healthcare organizations [28].

In response to the question regarding the “*6—Most frequently reported consequences for nurses affected by the second victim phenomenon*,” participants’ answers could be categorized as **intrapersonal** and **interpersonal** consequences.

**In terms of intrapersonal consequences**, nurses identified loss of confidence, vulnerability, tendencies toward substance abuse, disrupted sleep patterns, need for recovery time, reduced job satisfaction, weakened professional reputation, permanent emotional scars, increased burnout trajectory, and feelings of insecurity [19,33,34,38,46]. Hypervigilance is also described, with nurses reporting obsessive or over-controlled behaviors [18,36]. In more extreme cases, suicidal ideation was reported as a direct consequence of the second victim experience [12,35,38,46]. Nurses also describe feelings of *being alone* [31] and a diminished sense of self-efficacy [34].

**Regarding interpersonal consequences**, nurses reported professional isolation, which leads to professional paralysis and an inability to cope within the required occupational framework [38]. Nurses expressed feeling compelled to leave their career or quit the profession altogether [35], as well as experiencing turnover intentions [19,35] and absenteeism [19,35].

## 4. Discussion

The results of this SR confirm the growing attention paid to this phenomenon of second victims in Western nurses over the last decade, especially in the post-pandemic period, as reinforced by the substantial increase in publications after 2019 [4,6,7,9,10,11,12,13,14,15,16,17,19,20,22,26,27,28,29,30,31,32,34,35,37,41,42,44,45,47,49,50].

This trend has already been identified in the literature, reflecting a growing interest in understanding the impact of adverse events on health professionals, particularly nurses [12]. Across the included studies, findings consistently describe emotional, psychological, and behavioral responses following adverse events, with a recurring progression of distress and recovery phases. However, despite the reported presence of institutional support structures, nurses frequently perceive these resources as insufficient, difficult to access, or poorly aligned with their needs. This discrepancy suggests that organizational culture, managerial support, and systemic barriers may play a critical role in shaping nurses’ experiences and help-seeking behaviors after adverse events.

The predominance of primary studies, most of which are qualitative and cross-sectional, shows that the knowledge that exists is mainly descriptive and exploratory, and thus there are constraints in establishing causal relationships and developing interventions based on more robust evidence [12,14,17,29,33,35,37,41,42,43].

The diversity of practice settings identified, ranging from adult and pediatric inpatient units to intensive care units, emergency services, obstetrics and gynecology services, and operating theatres, confirms the cross-cutting nature of the second victim phenomenon [4,11,26,31,32,33,34,35,36,37,38]. However, the fact that many of the included documents fail to specify nurses’ practice contexts represents a significant barrier to identifying particularly vulnerable groups, limiting the ability to tailor prevention and support strategies to the specific demands of each setting [12,17,29,39,41,42,43,44,45,46]. This lack of contextual and organizational detail may also partially explain the persistent gap between the formal existence of institutional support structures and nurses’ reported perceptions of their limited accessibility or effectiveness. Consequently, insufficient contextual characterization can directly undermine institutions’ capacity to design, implement, and evaluate responsive and context-sensitive support mechanisms [40].

A clear discrepancy persists between the existence of institutional support structures and nurses’ perceptions of their accessibility and usefulness, suggesting systemic failures in implementation, communication, and organizational culture. Although several documents describe formal emotional support programs, protocols and structured resources [4,11,12,17,19,27,32,34,40,43,47], they are often unknown, perceived as insufficient or not used [32,41,42]. This contradiction demonstrates the persistence of a gap between institutional policies and actual practices in these contexts, demonstrating organizational communication failures as well as barriers, including the stigma associated with error and reprisals [33].

On the other hand, the review results show the importance of informal support, particularly peer support and support between family members and friends, as the main source of support reported by nurses [12,37]. These findings indicate that organizations must transition from reactive approaches to structured, proactive, and integrated support systems that do not depend on individual initiative. This predominance reinforces the absence of formal structures but also the importance these professionals attach to established relationships of trust, which promote emotional sharing and validation of the experience. However, the fact that professionals rely exclusively on informal support may limit their access to structured interventions recognized as facilitating recovery, namely peer support programs and institutional resources that reduce stress, promote coping and are central to recovery [7,26,27,28,34,51].

Organizations are responsible for safeguarding the protection of healthcare professionals by implementing measures that enhance working conditions and by fostering the integration of personal and professional support systems [4,11,12,17,19,27,32,34,40,43,47].

The wide range of symptoms reported [4,7,12,19,29,33,34,48], including emotional [12,35,38] and psychological [4,7,19,28,29,33,35,43,51] symptoms, reinforces the profound and multidimensional impact of this phenomenon on nurses. These findings suggest that the second victim phenomenon can take on characteristics similar to acute and post-traumatic stress disorders [28]. The reference to suicidal ideation and suicide situations related to adverse events [12,37,38,46] highlights the potential seriousness of the phenomenon and the urgent need for structured responses [47,49,50,51,52].

The results also reinforce the persistence of gaps in health training on this topic, described as the absence or insufficiency of content related to error, its management and support for professionals [26,28,37,52]. This training gap may be associated with the limited knowledge and use of response strategies following adverse events, reflected in reduced use of available institutional support mechanisms [12,28,29,32,37,38,42].

Finally, the personal consequences identified, namely loss of trust, professional isolation, intentions to leave, and absenteeism [19,34,35,38], have direct implications for nurse retention and patient safety, reinforcing the need to consider the phenomenon of second victims among nurses as an organizational and systemic problem and not just an individual one [11,45]. In the discussion, we emphasized organizational responsibility and patient safety culture, providing a clearer point of reference while maintaining the descriptive and mapping nature of the review. Overall, this scoping review shows that although the second victim phenomenon is increasingly recognized, organizational responses remain fragmented, inconsistently implemented, and often insufficient. Institutions still rely heavily on informal support networks rather than structured programs, despite clear evidence of the impact on professionals and patient safety.

Future research should prioritize longitudinal and interventional studies to evaluate the effectiveness of support programs, as descriptive research alone is insufficient to guide evidence-based organizational change. In parallel, institutions must implement policies that ensure adequate dissemination and equitable access to support mechanisms for all nurses, reducing barriers such as communication failures, a punitive organizational culture, unclear pathways for accessing support, concerns about confidentiality, and gaps in managerial training [26,28,29,37,38,52].

### Strengths and Limitations

This SR has several methodological strengths, particularly in terms of compliance with the JBI and PRISMA-ScR guidelines, the use of a comprehensive search strategy across multiple databases as supported by manual verification of references, and the recent update of the initial search. The inclusion of different types of documents, as well as a double-blind, independent review with conflict resolution by a third reviewer, reinforces credibility and reduces the risk of selection bias. However, there are some limitations that should be considered. The heterogeneity of the included studies (in terms of methodological design and clinical contexts) is an obstacle to direct comparisons. The lack of information on the professional background of participants in several studies limited the identification of groups at higher risk of vulnerability. Finally, the predominance of descriptive studies may have limited the ability to infer causal relationships or assess the relevance of support interventions.

## 5. Conclusions

This SR confirms that the second victim phenomenon represents a systemic organizational challenge rather than an individual failure. The findings suggest that the experience of nurses as second victims is a relevant and growing issue, with a significant increase in publications following the COVID-19 pandemic. These results help summarize evidence that can inform nurse managers and raise their awareness of the consequences and challenges faced by nurses in Western contexts when they become second victims.

It is therefore essential to regard nurses in this situation not merely as facing an individual difficulty but as part of a broader organizational problem that requires structured, accessible, and integrated responses. A notable discrepancy persists between the resources provided by institutions and nurses’ perceptions of their accessibility and usefulness, with considerable reliance on informal support, especially from peers, family, and friends.

The findings also underscore the urgent need for formalized and mandatory institutional policies that ensure consistent dissemination, implementation, and monitoring of available support mechanisms, thereby preventing critical consequences for professional well-being. The range of physical, emotional, and psychological symptoms reported highlights the multidimensional impact of the phenomenon and its implications for patient safety and professional retention.

## Figures and Tables

**Table 1:** Eligibility criteria.

PCC Framework	Inclusion Criteria	Exclusion Criteria
Population	Clinical nurses of both genders and all ages from all specialties	Nursing students; other health professionals; veterinary nurses
Concept	Studies addressing the “second victim,” “second victim phenomenon,” or “second victim syndrome,” focusing on the nurses who were involved in medical errors and the impact of that experience had on their professional and personal life	
Context	All papers focus on healthcare institutions or services involving nurses as second victims in Western countries	All papers reporting Eastern countries
Format	Primary studies (quantitative, qualitative, and mixed methods) and literature reviews were considered, as were reports and guidelines or other technical documents issued by professional regulatory bodies, professional associations, scientific societies, or other organizations with recognized authority and standing in the field of human resources or people management	

**Table 2:** Search strategy used in Medline via PubMed (5 September 2024).

Search Strategy
(“Second Victim”[tiab] OR “Second Victim Phenomenon”[tiab] OR “Second Victim Syndrome”[tiab] OR “second victims”[tiab]) AND (“Nurses”[MeSH] OR “nurse”[tiab] OR “nurses”[tiab]) AND (“Western countries”[tiab] OR “Western context”[tiab] OR “Europe”[tiab] OR “North America”[tiab] OR “United States”[tiab] OR “Canada”[tiab] OR “Australia”[tiab] OR “New Zealand”[tiab]) (“Second Victim”[Title/Abstract] OR “Second Victim Phenomenon”[Title/Abstract] OR “Second Victim Syndrome”[Title/Abstract] OR “second victims”[Title/Abstract]) AND (“Nurs*”[MeSH Terms] OR “nurse”[Title/Abstract] OR “Nurses”[Title/Abstract]) AND (“Western countries”[Title/Abstract] OR “Western context”[Title/Abstract] OR “Europe”[Title/Abstract] OR “North America”[Title/Abstract] OR “United States”[Title/Abstract] OR “Canada”[Title/Abstract] OR “Australia”[Title/Abstract] OR “New Zealand”[Title/Abstract] OR “Israel”[Title/Abstract])).

MH—MeSH terms; * truncation.

## Data Availability

No new data were created or analyzed in this study.

## Supplementary Materials

The following supporting information can be downloaded at: https://www.mdpi.com/article/10.3390/healthcare14040467/s1, File S1: PRISMA-ScR Checklist; Table S2: Summary of the included studies. Reference [58] is cited in the Supplementary Materials.

## Abbreviations

The following abbreviations are used in this manuscript:

JBI	Joanna Briggs Institute
SR	Scoping Review

## References

[1] Ozeke O., Ozeke V., Coskun O., Budakoglu I.I. (2019). Second victims in health care: Current perspectives. Adv. Med. Educ. Pract..

[2] Makary M.A., Daniel M. (2016). Medical error-the third leading cause of death in the US. BMJ.

[3] Kohn L.T., Corrigan J.M., Donaldson M.S. (2000). To Err Is Human: Building a Safer Health System.

[4] Thompson M., Hunnicutt R., Broadhead M., Vining B., Aroke E.N. (2022). Implementation of a certified registered nurse anesthetist second victim peer support program. J. PeriAnesth. Nurs..

[5] Wu A.W. (2000). Medical error: The second victim: The doctor who makes the mistake needs help too. BMJ.

[6] Huang H., Liu T., Peng Y., Du X., Huang Q., Zhao Q., Xiao M., Luo Y., Zheng S. (2024). “Learn from Errors”: Post-traumatic growth among second victims. BMC Public Health.

[7] Sahay A., McKenna L. (2023). Nurses and nursing students as second victims: A scoping review. Nurs. Outlook.

[8] Ong T., Goh C., Tan E., Sivanathan K., Tang A., Tan H., Ng Q.X. (2025). Second victim syndrome among healthcare professionals: A systematic review of interventions and outcomes. J. Healthc. Leadersh..

[9] Naya K., Aikawa G., Ouchi A., Ikeda M., Fukushima A., Yamada S., Sakuramoto H. (2023). Second victim syndrome in intensive care unit healthcare workers: A systematic review and meta-analysis on types, prevalence, risk factors, and recovery time. PLoS ONE.

[10] Catalán L., Kappes M., Morgado G., Oliveira D. (2024). Ethical issues in research with second victims: A scoping review. Nurs. Ethics.

[11] Hess A., Flicek T., Watral A.T., Phillips M., Derby K., Ayres S., Carney J., Voll A., Blocker R. (2024). BONE break: A hot debrief tool to reduce second victim syndrome for nurses. Jt. Comm. J. Qual. Patient Saf..

[12] Strametz R., Fendel J.C., Koch P., Roesner H., Zilezinski M., Bushuven S., Raspe M. (2021). Prevalence of second victims, risk factors, and support strategies among German nurses (SeViD-II Survey). Int. J. Environ. Res. Public Health.

[13] New L., Lambeth T. (2024). Second victim phenomenon. Nurs. Clin. N. Am..

[14] Kruse J.A., Podojil-Kostecki P., Smith B. (2024). Living with the aftermath: The second victim experience among certified registered nurse anesthetists. AANA J..

[15] Shao Y., Li S., Wei L., Shan X., Zhou D., Zhang Y., Wei H. (2023). Nurses’ second victim experience, job insecurity, and turnover intention: A latent profile analysis. Res. Nurs. Health.

[16] Shao Y., Shan X., Li S., Zhang X., Chi K., Xu Y., Wei H. (2024). Mediating role of rumination in second victim experience to turnover intention in psychiatric nurses. Nurs. Res..

[17] Alevi J., Mira J., Bohomol E., Draganov P., Domenico E. (2024). The newly graduated nurse as a second victim. Acta Paul. Enferm..

[18] Mak S., Thomas A. (2022). Steps for conducting a scoping review. J. Grad. Med. Educ..

[19] Cohen R., Sela Y., Hochwald I.H., Nissanholz-Gannot R. (2023). Nurses’ silence: Understanding the impacts of second victim phenomenon among Israeli nurses. Healthcare.

[20] Sánchez-García A., Saurín-Morán P.J., Carrillo I., Tella S., Põlluste K., Srulovici E., Buttigieg S.C., Mira J.J. (2023). Patient safety topics, especially the second victim phenomenon, are neglected in undergraduate medical and nursing curricula in Europe: An online observational study. BMC Nurs..

[21] Fujita S., Seto K., Ito S., Wu Y., Huang C.C., Hasegawa T. (2013). The characteristics of patient safety culture in Japan, Taiwan and the United States. BMC Health Serv. Res..

[22] Kang S., Ho T.T.T., Lee N.J. (2021). Comparative studies on patient safety culture to strengthen health systems among Southeast Asian countries. Front. Public Health.

[23] Aromataris E., Lockwood C., Porritt K., Pilla B., Jordan Z. (2024). JBI Manual for Evidence Synthesis North Adelaide: JBI.

[24] Peters M.D.J., Marnie C., Colquhoun H., Garritty C.M., Hempel S., Horsley T., Langlois E.V., Lillie E., O’bRien K.K., Tunçalp Ö. (2021). Scoping reviews: Reinforcing and advancing the methodology and application. Syst. Rev..

[25] Davies E.L., Pollock D., Graham A., Laing R.E., Langton V., Bulto L., Kelly J. (2022). Reporting of patient journey mapping in current literature: A scoping review protocol. JBI Evid. Synth..

[26] Davis A.E., Copeland-Streeter D.J., Okoye R.R. (2025). Effects of a second victim peer support program in the pediatric intensive care unit. Am. J. Nurs..

[27] Peddle M., McPhillips M., Cross R., Zarb L. (2025). Experiences and support of Australian nurses who identify as a second victim: A mixed methods study. Appl. Nurs. Res..

[28] Fisher S., Blau A., Gendler Y. (2025). The silent struggle: An integrative review of PTSD symptoms in second victim experiences among nurses. Int. Nurs. Rev..

[29] Istrate M.I., Forray A.I., Ungureanu M.I., Mira J.J., Constantinescu S.A., Cherecheș R.M. (2025). Assessing safety culture and second victim experience following adverse events among Romanian nurses: A cross-sectional study. BMC Nurs..

[30] Khosravi A., Mahat S., Syyrilä T., Härkänen M. (2025). Negative emotions experienced on the occurrence of medication errors by nurses: A mixed-method systematic review. J. Clin. Nurs..

[31] Aharon A.A., Fariba M., Shoshana F., Melnikov S. (2021). Nurses as second victims to their patients’ suicidal attempts: A mixed-method study. J. Clin. Nurs..

[32] Quadros D.V., de Magalhães A.M.M., Boufleuer E., Tavares J.P., de Souza Kuchenbecker R., Pai D.D. (2022). Falls suffered by hospitalized adult patients: Support to the nursing team as the second victim. Aquichan.

[33] Quillivan R.R., Burlison J.D., Browne E.K., Scott S.D., Hoffman J.M. (2016). Patient safety culture and the second victim phenomenon: Connecting culture to staff distress in nurses. Jt. Comm. J. Qual. Patient Saf..

[34] Ganahl S., Knaus M., Wiesenhuetter I., Klemm V., Jabinger E.M., Strametz R. (2022). Second victims in intensive care: Emotional stress and traumatization of intensive care nurses in western Austria after adverse events during the treatment of patients. Int. J. Environ. Res. Public Health.

[35] Kappes M., Delgado-Hito P., Contreras V.R., Romero-García M. (2023). Prevalence of the second victim phenomenon among intensive care unit nurses and the support provided by their organizations. Nurs. Crit. Care.

[36] Kappes M., Romero-Garcia M., Sanchez M., Delgado-Hito P. (2024). Coping trajectories of intensive care nurses as second victims: A grounded theory. Aust. Crit. Care.

[37] Finney R.E., Torbenson V.E., Riggan K.A., Weaver A.L., Long M.E., Allyse M.A., Rivera-Chiauzzi E.Y. (2020). Second victim experiences of nurses in obstetrics and gynaecology: A Second Victim Experience and Support Tool Survey. J. Nurs. Manag..

[38] Daniels R., McCorke R. (2016). Design of an evidence-based second victim curriculum for nurse anesthetists. AANA J..

[39] Burlison J.D., Scott S.D., Browne E.K., Thompson S.G., Hoffman J.M. (2017). The second victim experience and support tool: Validation of an organizational resource for assessing second victim effects and the quality of support resources. J. Patient Saf..

[40] Cabilan C.J., Kynoch K. (2017). Experiences of and support for nurses as second victims of adverse nursing errors: A qualitative systematic review. JBI Database Syst. Rev. Implement. Rep..

[41] Connors C.A., Dukhanin V., March A.L., Parks J.A., Norvell M., Wu A.W. (2019). Peer support for nurses as second victims: Resilience, burnout, and job satisfaction. J. Patient Saf. Risk Manag..

[42] Draus C., Mianecki T.B., Musgrove H., Bastien D.J., Greggs D., Halash C., Lewis A.B., Mackenzie W.M. (2022). Perceptions of nurses who are second victims in a hospital setting. J. Nurs. Care Qual..

[43] Hinkley T.L. (2022). The combined effect of psychological and social capital in registered nurses experiencing second victimization: Aa structural equation model. J. Nurs. Scholarsh..

[44] Järvisalo P., Haatainen K., Von Bonsdorff M., Turunen H., Härkänen M. (2024). Interventions to support nurses as second victims of patient safety incidents: A qualitative study of nurse managers’ perceptions. J. Adv. Nurs..

[45] Moran D., Wu A.W., Connors C., Chappidi M.R., Sreedhara S.K., Selter J.H., Padula W.V. (2020). Cost-benefit analysis of a support program for nursing staff. J. Patient Saf..

[46] Stone M. (2020). Second victim support: Nurses’ perspectives of organizational support after an adverse event. J. Nurs. Adm..

[47] Martins R., Oliveira A., Sobrinho N., Santos A., Batista J. (2023). Ações de apoio à enfermagem envolvida como segunda vítima de erros e eventos adversos: Revisão interativa. Varia Sci. Cienc. Saude.

[48] Busch I.M., Moretti F., Purgato M., Barbui C., Wu A.W., Rimondini M. (2020). Psychological and psychosomatic symptoms of second victims of adverse events: A systematic review and meta-analysis. J. Patient Saf..

[49] Mira J., Carillo I., Tella S., Vanhaecht K., Panella M., Seys D., Ungureanu M.-I., Sousa P., Buttigieg S.C., Vella-Bonanno P. (2024). The European Researchers’ Network Working on Second Victim (ERNST) policy statement on the second victim phenomenon for increasing patient safety. Public Health Rev..

[50] Pimenta I.R. (2021). Apoio aos Profissionais de Saúde: A Exposição a Incidentes de Segurança do Doente e a Existência de Estruturas de Suporte. Master’s Thesis.

[51] Busch I.M., Moretti F., Campagna I., Benoni R., Tardivo S., Wu A.W., Rimondini M. (2021). Promoting the psychological well-being of healthcare providers facing the burden of adverse events: A systematic review of second victim support resources. Int. J. Environ. Res. Public Health.

[52] Directorate-General of Health [DGS] (2023). Documento Técnico para a Implementação do Plano Nacional para a Segurança dos Doentes 2021–2026.

[53] Research Group on Second and Third Victims (2015). Recommendations for Providing an Appropriate Response When Patients Experience an Adverse Event with Support for Healthcare’s Second and Third Victims.

[54] Vanhaecht K., Seys D., Russotto S., Strametz R., Mira J., Sigurgeirsdóttir S., Wu A.W., Põlluste K., Popovici D.G., Sfetcu R. (2022). An Evidence and Consensus-Based Definition of Second Victim: A Strategic Topic in Healthcare Quality, Patient Safety, Person-Centeredness and Human Resource Management. Int. J. Environ. Res. Public Health.

[55] World Health Organization [WHO] (2021). Global Patient Safety Action Plan 2021–2030: Towards Eliminating Avoidable Harm in Health Care.

[56] Bleazard M. (2019). Clinical Nurse Specialist Practice Interventions for Second Victims of Adverse Patient Events. Clin. Nurse Spec..

[57] Order No. 9390/2021 approving the National Plan for Patient Safety 2021–2026. *Diário da Républica Portuguesa*, 24 September 2021; II Série (185). https://files.diariodarepublica.pt/2s/2021/09/187000000/0009600103.pdf.

[58] Tricco A.C., Lillie E., Zarin W., O’Brien K.K., Colquhoun H., Levac D., Moher D., Peters M.D., Horsley T., Weeks L. (2018). PRISMA Extension for Scoping Reviews: Checklist and Explanation. Ann Intern Med..

