# Supplementary material for: Mapping the Second Victim Experience Among Western Nurses: A Scoping Review
Source: Healthcare (Basel). 2026 Feb 12;14(4):467. doi: 10.3390/healthcare14040467 (PMC12940687; doi:10.3390/healthcare14040467)
Supplement: Supplementary file 1 [file healthcare-14-00467-s001.zip › healthcare-4096827-supplementary.pdf]

Supplementary File S1

S1: Preferred Reporting Items for Systematic reviews and Meta-Analyses extension for Scoping Reviews (PRISMA-ScR) Checklist [58]

SECTION	ITEM	PRISMA-ScR CHECKLIST ITEM	REPORTED ON PAGE
TITLE			
Title	1	Identify the report as a scoping review.	1
ABSTRACT			
Structured summary	2	Provide a structured summary that includes (as applicable) background, objectives, eligibility criteria, sources of evidence, charting methods, results, and conclusions that relate to the review questions and objectives.	1
INTRODUCTION			
Rationale	3	Describe the rationale for the review in the context of what is already known. Explain why the review questions/objectives lend themselves to a scoping review approach.	2
Objectives	4	Provide an explicit statement of the questions and objectives being addressed with reference to their key elements (e.g., population or participants, concepts, and context) or other relevant key elements used to conceptualize the review questions and/or objectives.	2
METHODS			
Protocol and registration	5	Indicate whether a review protocol exists; state if and where it can be accessed (e.g., a Web address); and if available, provide registration information, including the registration number.	3
Eligibility criteria	6	Specify characteristics of the sources of evidence used as eligibility criteria (e.g., years considered, language, and publication status), and provide a rationale.	3-4
Information sources*	7	Describe all information sources in the search (e.g., databases with dates of coverage and contact with authors to identify additional sources), as well as the date the most recent search was executed.	4
Search	8	Present the full electronic search strategy for at least 1 database, including any limits used, such that it could be repeated.	4
Selection of sources of evidence†	9	State the process for selecting sources of evidence (i.e., screening and eligibility) included in the scoping review.	4-5
Data charting process‡	10	Describe the methods of charting data from the included sources of evidence (e.g., calibrated forms or forms that have been tested by the team before their use, and whether data charting was done independently or in duplicate) and any processes for obtaining and confirming data from investigators.	5

SECTION	ITEM	PRISMA-ScR CHECKLIST ITEM	REPORTED ON PAGE
Data items	11	List and define all variables for which data were sought and any assumptions and simplifications made.	5-6
Critical appraisal of individual sources of evidence§	12	If done, provide a rationale for conducting a critical appraisal of included sources of evidence; describe the methods used and how this information was used in any data synthesis (if appropriate).	*Not applicable*
Synthesis of results	13	Describe the methods of handling and summarizing the data that were charted.	6
RESULTS			
Selection of sources of evidence	14	Give numbers of sources of evidence screened, assessed for eligibility, and included in the review, with reasons for exclusions at each stage, ideally using a flow diagram.	7-10
Characteristics of sources of evidence	15	For each source of evidence, present characteristics for which data were charted and provide the citations.	7-10
Critical appraisal within sources of evidence	16	If done, present data on critical appraisal of included sources of evidence (see item 12).	Not applicable
Results of individual sources of evidence	17	For each included source of evidence, present the relevant data that was charted that relates to the review questions and objectives.	7-10 and Suplementary File S2
Synthesis of results	18	Summarize and/or present the charting results as they relate to the review questions and objectives.	6-9 and Suplementary File S2
DISCUSSION			
Summary of evidence	19	Summarize the main results (including an overview of concepts, themes, and types of evidence available), link to the review questions and objectives, and consider the relevance to key groups.	10-12
Limitations	20	Discuss the limitations of the scoping review process.	11-12
Conclusions	21	Provide a general interpretation of the results with respect to the review questions and objectives, as well as potential implications and/or next steps.	13
FUNDING			
Funding	22	Describe sources of funding for the included sources of evidence, as well as sources of funding for the scoping review. Describe the role of the funders of the scoping review.	11

Supplementary File S2

Table S2: Summary of the included studies

Authors and year of publication	Title	Country	Type of study/ Type of Document	Objective(s)	Data collecting / Sample/ Setting	Main Outcomes
(Aharon et al., 2021) [31]	Nurses as 'second victims' to their patients' suicidal attempts: A mixed-method study	Israel	Mixed-methods study with a sequential exploratory approach.	(1) To understand the effects of patients' suicidal attempts and events on nurses' second victim symptoms and to explore the association between these experiences and nurse absenteeism and turnover.	150 nurses who worked in internal departments. The Second Victim Experience and Support Tool were used to substantiate and measure second victim related distress of nurses who treated suicidal patients.	The qualitative part identified three themes and nine sub-themes, including the new variable 'sense of being alone'. The quantitative part of the study found that nurses expressed a medium level of second victim related distress. After controlling demographic variables, second victim distress and the sense of being alone following patients' suicidal events may explain nurse absenteeism and turnover.
(Alevi et al., 2024) [17]	The newly graduated nurse as a second victim	Brazil	Cross-sectional, descriptive study with a quantitative approach.	(1) To describe the prevalence of newly graduated nurses as secondary victims of adverse events<br>(2) To understand the support conditions, they receive in healthcare institutions.	n=138 newly graduated nurses who agreed to answer the online questionnaire.	"The prevalence of newly graduated nurses involved in adverse events was 26.8%, and among those who experienced such an incident, the majority reported negative feelings and insecurity in their work as an outcome. After the event, the support received mostly came from colleagues and significant others, and regarding institutional support, the need for emotional support programs is highlighted so that these professionals can

Authors and year of publication	Title	Country	Type of study/ Type of Document	Objective(s)	Data collecting / Sample/ Setting	Main Outcomes
						overcome the challenges of being a second victim".
(Bleazard, 2019) [56]	Clinical Nurse Specialist Practice Interventions for Second Victims of Adverse Patient Events.	USA	Narrative review / practice recommendations	(1) To provide clinical nurse specialists a summary of: second-victim impact secondary to adverse patient events; a review of associated negative outcomes; and clinical nurse specialist practice recommendations.	Evidence published.	"Clinical nurse specialist organization/system interventions to reduce the consequences of second-victim impact include supporting a culture of safety, leading organizational improvement, as well as advocating for education and support for second victims on the national level".
(Burlison, 2017) [39]	The Second Victim Experience and Support Tool: Validation of an Organizational Resource for Assessing Second Victim Effects and the Quality of Support Resources	USA	Methodological Study	(1) To present the development and psychometric evaluation of the Second Victim Experience and Support Tool (SVEST), a survey instrument that can assist health care organizations to implement and track the performance of second victim support resources.	The SVEST (29 items representing 7 dimensions and 2 outcome variables) was completed by 303 health care providers involved in direct patient care.	"The survey collected responses on second victim-related psychological and physical symptoms and the quality of support resources. Desirability of possible support resources was also measured. The SVEST was assessed for content validity, internal consistency, and construct validity with confirmatory factor analysis".
(Busch, 2020) [48]	Psychological and Psychosomatic Symptoms of Second Victims of Adverse Events: a Systematic	Italy	Systematic Review and Meta-Analysis	(1) To review systematically the types and prevalence of psychological and psychosomatic symptoms among second victims.	Nine electronic databases up to February 2017.	"Second victims report a high prevalence and wide range of psychological symptoms. More than two-thirds of providers reported troubling memories, anxiety, anger, remorse, and distress. Preventive and therapeutic

Authors and year of publication	Title	Country	Type of study/ Type of Document	Objective(s)	Data collecting / Sample/ Setting	Main Outcomes
	Review and Meta-Analysis"					programs should aim to decrease second victims' emotional".
(Busch, 2021) [51]	Promoting the Psychological Well-Being of Healthcare Providers Facing the Burden of Adverse Events: A Systematic Review of Second Victim Support Resources	Italy	Literature Review	(1) To describe the types of support resources available in healthcare organizations, their benefits for second victims, peer supporters' experiences, and implementation challenges.	Six databases up to 19 December 2019 and additional literature, including weekly search alerts until 21 January 2021	Investing in second victim support structures should be a top priority for healthcare institutions adopting a systemic approach to safety and striving for just culture.
(Cabilan & Kynoch, 2017) [40]	Experiences of and support for nurses as second victims of adverse nursing errors: a qualitative systematic review	Australia	Qualitative systematic Review	(1) To synthesize the best available evidence on nurses' experiences as second victims, and explore their experiences of the support they receive and the support they need.	Nine qualitative studies included in the review. The narratives of 284 nurses generated a total of 43 findings, which formed 15 categories based on similarity of meaning	"Adverse errors were distressing for nurses, but they did not always receive the support they needed from colleagues. The lack of support had a significant impact on nurses' decisions on whether to disclose the error and his/her recovery process. Therefore, a good support system is imperative in alleviating the emotional burden, promoting the disclosure process, and assisting nurses with reconciliation. This review also highlighted research gaps that encompass the characteristics of the support system preferred by nurses, and the scarcity of studies worldwide".

Authors and year of publication	Title	Country	Type of study/ Type of Document	Objective(s)	Data collecting / Sample/ Setting	Main Outcomes
(Cohen et al., 2023) [19]	Nurses' Silence: Understanding the Impacts of Second Victim Phenomenon among Israeli Nurses	Israel	Descriptive Qualitative Approach	(1) To examine the impact of the second victim on Israeli nurses, with a specific focus on the organizational support they felt they required compared with the support they felt that they had received from their organizations.	n=15 Israeli nurses/ In-depth interviews were conducted, using a semi-structured questionnaire, among nurses who had experienced the SVP (second victim phenomenon). December 2022 and February 2023	"Appropriate organizational support, offered proximal to an adverse event as well as over time, is essential for the nurse, the patient, and the organization. Personal barriers, together with limited awareness, may challenge the identification and provision of appropriate assistance. Hence, it is important to address the phenomenon as part of the general organizational policy to improve the quality of care and patient safety".
(Connors et al, 2019) [41]	Peer support for nurses as second victims: Resilience, burnout, and job satisfaction.	USA	Cross-sectional surveys	(1) To evaluate awareness and utilization of Resilience in Stressful Events among nurses at one teaching hospital; perceptions of program benefits; resilience, burnout, and job satisfaction among Resilience in Stressful Events users versus non-users.	Staff nurses and nurse leaders (n= 337) / surveys	"Nurses indicated favorable perceptions of using Resilience in Stressful Events, and its utilization was associated with greater resilience but higher burnout in frontline nurses".
(Daniels & McCorkle, 2016) [38]	Design of an Evidence-Based "Second Victim" Curriculum for Nurse Anesthetists	USA	Literature Review	(1) To identify content for an educational program on second victim for nurse anesthetists through a systematic review of scientific literature.	Search engines: Orbis Yale University Catalog, CINAHL, Pub Med, Ovid, Cochrane Library, EBSCO Databases, and Google Scholar, between	The second victim phenomenon presents substantial challenges for healthcare professionals, making it essential to acknowledge its occurrence and develop an evidence-based educational curriculum that supports the creation of peer and organizational

Authors and year of publication	Title	Country	Type of study/ Type of Document	Objective(s)	Data collecting / Sample/ Setting	Main Outcomes
				(2) To validate the content for an education program on second victimhood using a panel of experts on second victim in healthcare.	February 7, 2014, through August 9, 2014. (n=24)	support structures to provide adequate assistance after adverse events.
(Davis et al., 2025) [26]	Effects of a Second Victim Peer Support Program in the Pediatric Intensive Care Unit	USA	Quality Improvement Project	(1) Reducing distress levels among second victims after a stressful patient-related event	Participants (nurses) (n = 13) measured their distress levels on a distress scale both before and after peer support team encounters.	"Nurse leaders are well-positioned to lead the development of support programs that promote resilience and the well-being of nurses and other health care professionals in mitigating the adverse effects of second victim syndrome".
(Directorate-General of Health [DGS], 2022) [52]	*Documento Técnico para a Implementação do Plano Nacional para a Segurança dos Doentes (PNSD) 2021-2026* (Technical Document for the Implementation of the National Plan for Patient Safety (PNSD) 2021–2026)	Portugal	Technical Document to Best practice	(1) To serve as a facilitating tool for the implementation process of the PNSD 2021–2026, as well as to support aspects of its operationalization and lay the foundations for uniform execution at the national level and for the achievement of its goals and objectives, while facilitating the work of all healthcare professionals, patient-safety managers, and leadership teams.	Not applicable	Describe the tools and strategies that support the implementation of the National Plan for Patient Safety 2021–2026 in clinical settings. It is crucial to implement preventive measures, avoiding clinical errors.

Authors and year of publication	Title	Country	Type of study/ Type of Document	Objective(s)	Data collecting / Sample/ Setting	Main Outcomes
(Draus et al., 2022) [42]	Perceptions of Nurses Who Are Second Victims in a Hospital Setting	USA	Descriptive study	(1) To determine the prevalence of nurses who identified as Second Victims (SVs) and their awareness and use of supportive resources.	159 nurses as SVs / survey	"Adverse events trigger emotional trauma in SVs who require administrative awareness, support, and follow-up to minimize psychological trauma in the clinical nurse".
(Finney et al., 2020) [37]	Second victim experiences of nurses in obstetrics and gynecology: A Second Victim Experience and Support Tool Survey	USA	Descriptive (quantitative)	(1) To investigate second victim experiences and supportive resources for nurses in obstetrics and gynecology.	n=115 nurses in obstetrics and gynecology (Nurses at a single institution) / survey from 1st July to 1st September 2019.	"Nurses in Obstetrics and Gynecology face clinical and non-clinical situations that lead to potential second victim experiences. Educational opportunities and peer supportive interventions specific to second victim experiences should be encouraged".
(Fisher et al., 2025) [28]	The Silent Struggle: An Integrative Review of PTSD Symptoms in Second Victim Experiences Among Nurses	Israel	Comprehensive Integrative Review	(1) To explore the parallels between second victim symptoms and post-traumatic stress disorder, emphasizing the need for a better understanding of their intersection	58 titles and including 19 studies examining the second victim's emotional and psychological consequences.	"Effective support systems, including targeted training, institutional policies, and integrated mental health programs, are essential to mitigate the impact of second victim experiences. Policymakers should prioritize the implementation of standardized peer support frameworks and resilience training within healthcare organizations. Further research is needed to refine these interventions and establish evidence-based policies that provide long-term psychological support for affected healthcare professionals".
(Ganahl et al, 2022) [34]	Second Victims in Intensive Care-Emotional Stress and	Austria	Qualitative research approach	(1) To evaluate the natural history and cause of second victim traumatization in	n=20 intensive care nurses in Western Austria. / Guided semi-	Intensive care nurses are exposed to many exceptional situations which have a high likelihood of resulting in second victim

Authors and year of publication	Title	Country	Type of study/ Type of Document	Objective(s)	Data collecting / Sample/ Setting	Main Outcomes
	Traumatization of Intensive Care Nurses in Western Austria after Adverse Events during the Treatment of Patients			Western Austria for the first time to tailor specific intervention.	structured interview (guide was developed based on the content-related topics and research questions by combining trial interviews with literature research on the topic of second victim).	traumatization. As proximal psychosocial support is a main source of coping, widespread implementation of effective psychosocial peer support programs ought to be applied by medical organizations. Patient safety measures such as proactive and reactive clinical risk management (e.g., CIRS) should be linked to second victim support.
(Hess et al., 2024) [11]	BONE Break: A Hot Debrief Tool to Reduce Second Victim Syndrome for Nurses	USA	Quality Improvement Project	(2) To understand the support conditions, they receive in healthcare institutions.	Emergency Department (ED) of a 450-bed urban acute-care hospital in the United States. The ED sees approximately 65,000 patient visits annually and employs 65 registered nurses with varying levels of experience.	During its initial implementation, BONE Break was employed in 43 of 46 events adverse events (93.5%), and 41 of 43 sessions (95.3%) were deemed helpful. The research team has continued to gain stakeholder buy-in and implement BONE Break across multiple sites. Future work will determine BONE Break's efficacy in enhancing long-term nursing retention and reducing second victim symptoms.
	The combined effect of psychological and social capital in registered nurses experiencing second victimization: A structural equation model	USA	Ex post facto, cross-sectional, non-experimental survey design.	(1) To examine the combined role psychological capital and social capital play in the severity of second victim syndrome experienced by registered nurses.	1167 nurses recruited through 12 professional nursing associations in the United States / Self-report questionnaires were administered to measure psychological	"Programmatic efforts should also focus on social capital at the team level as well as the importance of building self-efficacy through increasing mastery experiences, modeling of behavior, social persuasion and monitoring one's physiological responses".

Authors and year of publication	Title	Country	Type of study/ Type of Document	Objective(s)	Data collecting / Sample/ Setting	Main Outcomes
(Hinkley, 2022) [43]					capital (Psychological Capital Questionnaire), social capital (Social Capital Outcomes for Nurses) and second victim syndrome (Second Victim Experience and Support Tool).	
(Istrate et al., 2025) [29]	Assessing safety culture and second victim experience following adverse events among Romanian nurses: a cross-sectional study	Romania	Cross-sectional Study	(1) To examine their experiences within the patient safety culture and the psychological consequences of AEs (Healthcare adverse events)	n= 995 nurses/ Surveys.	"The significant emotional and professional impact of AEs on nurses in Romania, highlighting ongoing challenges in healthcare environments. The positive perception of safety culture among nurses suggests a basis for improvement, while training needs underscore areas for intervention. Tackling the second victim phenomenon is crucial for maintaining patient safety".
(Järvisalo et al., 2024) [44]	Interventions to support nurses as second victims of patient safety incidents: A qualitative study of nurse managers' perceptions.	Finland	Qualitative Study	(1) To describe nurse managers' perceptions of interventions to support nurses as second victims of patient safety incidents and to describe the management of interventions and ways to improve them.	Nurse managers (n = 16) recruited from three hospital districts in Finland was interviewed in 2021.	The study identified three main categories: (1) Management of second victim support, which contained three sub-categories related to the nurse manager's role, support received by the nurse manager and challenges of support management; (2) interventions to support second victims included existing interventions and operating models; and (3) improving second victim support, based on the

Authors and year of publication	Title	Country	Type of study/ Type of Document	Objective(s)	Data collecting / Sample/ Setting	Main Outcomes
						sub-categories developing practices and developing an open and non-blaming patient safety culture.
(Kappes et al., 2023) [35]	Prevalence of the second victim phenomenon among intensive care unit nurses and the support provided by their organizations	Chile	Multicenter, quantitative, cross-sectional and correlational study	(1) To determine the prevalence of second victimhood, focusing on psychological distress, among Chilean adult intensive care nurses and its relationship with the support provided by their organizations.	Intensive Care Unit (ICU) Nurses (n=326 nurses who been working in the adult ICU in Chile for more than 6 months/ survey with Three validated Instruments.	"Two-thirds of Chilean adult intensive care unit nurses report psychological stress following an adverse event. These results should be assessed internationally because second victims have major implications for the well-being of health professionals and, therefore, for retention and the quality of care. Critical care leaders must actively promote a safe environment for learning from adverse events, and hospitals must establish a culture of quality that includes support programs for second victims".
(Kapples et al., 2024) [35]	Coping trajectories of intensive care nurses as second victims: A grounded theory	Chile	Qualitative research through the grounded theory method	(1) To describe the coping trajectories of second victims among nurses working in ICUs (Intensive Care Units) in public hospitals in Chile	n=11 nurses working in Intensive care Units. Developed in-depth interviews conducted between March and May 2023, as well as a focus group interview.	For the coping process of ICU (Intensive Care Units) nurses following an adverse error, the most crucial factor is the support from colleagues and supervisors.
(Khosravi et al., 2025) [30]	Negative Emotions Experienced on the Occurrence of	Finland	Systematic Review	(1) To explore the negative emotions experienced by	1619 screened studies, from: PubMed, Scopus, Web of Science,	"The negative emotions that nurses experience as second victims can persist long after the error occurs. It underscores the need for

Authors and year of publication	Title	Country	Type of study/ Type of Document	Objective(s)	Data collecting / Sample/ Setting	Main Outcomes
	Medication Errors by Nurses: A Mixed-Method Systematic Review			nurses following medication errors.	Cumulative Index to Nursing and Allied Health Literature, PsycINFO and Google Scholar for studies published in English between January 2013 and October 2024	structured psychological support systems to foster a culture of 'responsibility without blame'".
(Kruse et al., 2024) [14]	Living With the Aftermath: The Second Victim Experience Among Certified Registered Nurse Anesthetists	USA	Descriptive Cross-Sectional Survey	(1) To understand certified registered nurse anesthetists' (CRNAs') second victim distress, perceived support, and the impact of the second victim experience on absenteeism and turnover intention.	CRNAs from the Michigan Association of Nurse Anesthetists, with a total of 172 / Survey.	"Turnover intentions and absenteeism were also evaluated with 11.6% of CRNAs wanting to take a job outside of patient care and/or quit their job, and 13% identified that they needed a mental health day and/or time away from work after their experience. Organizations must consider offering peer support and supportive counselling for practitioners who have suffered from traumatic events and identify desired forms of support among staff".
(Martins et al., 2023) [47]	*Ações de apoio à enfermagem envolvida como segunda vítima de erros e eventos adversos: Revisão integrative* (Support actions for nursing staff involved	Brazil	Integrative review	(1) To identify, in the literature, the support actions for nursing professionals involved as secondary victims of errors and adverse events.	Medical Literature Analysis and Retrieval System Online, Literatura Latino-Americana e do Caribe em Ciências da Saúde, Índice	Support actions for nursing staff involved as second victims occur mainly through dialogue between peers and institutional leaders. It is necessary to expand the discussion and implement systematized support programs in health services to strengthen

Authors and year of publication	Title	Country	Type of study/ Type of Document	Objective(s)	Data collecting / Sample/ Setting	Main Outcomes
	as second victims of errors and adverse events: Integrative review)				Bibliográfico Español en Ciencias de la Salud e Base de dados de Enfermagem, available by portal da Biblioteca Virtual da Saúde, during September 2023.	the organizational safety culture.
(Mira et al., 2024) [49]	The European Researchers' Network Working on Second Victim (ERNST) Policy Statement on the Second Victim Phenomenon for Increasing Patient Safety	Spain	Policy Brief	(1) To Inform policy	*Not applicable*	"Addressing the second victim phenomenon is essential for ensuring patient safety. By implementing supportive policies and fostering a just culture, healthcare systems can better manage the repercussions of AEs and support the wellbeing of healthcare professionals".
(Moran, 2020) [45]	Cost-Benefit Analysis of a Support Program for Nursing Staff	USA	Impact Project Markov model with a 1-year time horizon was developed	(1) To evaluate the impact of the Resilience In Stressful Events (RISE) program by conducting an economic evaluation of its cost benefit (2) To compare the cost benefit with and without the Resilience In Stressful Events (RISE) program from a provider (hospital) perspective	Nursing staff who used the RISE program between 2015 and 2016 at a 1000-bed, private hospital in the United States were included in the analysis. The cost of running the RISE program, nurse turnover, and nurse time off were modelled. Data on costs	"The RISE program resulted in substantial cost savings to the hospital. Hospitals should be encouraged by these findings to implement institution-wide support programs for medical staff, based on a high demand for this type of service and the potential for cost savings".

Authors and year of publication	Title	Country	Type of study/ Type of Document	Objective(s)	Data collecting / Sample/ Setting	Main Outcomes
					were obtained from literature review and hospital data. Probabilities of quitting or taking time off with or without the RISE program were estimated using survey data.	
(Order No. 9390/2021 approving the National Plan for Patient Safety 2021-2026, 2021) [57]	*Despacho n.º 9390/2021 que Aprova o Plano Nacional para a Segurança dos Doentes 2021-2026* (Order No. 9390/2021 approving the National Plan for Patient Safety 2021-2026)	Portugal	Legal Document	(1) To consolidate and promote safety in the provision of healthcare within the health system, particularly in the context of the direct delivery of care to patients	*Not applicable*	(1) This plan represents a milestone for the Portuguese health system in placing patient safety as a structured and systematic priority. (2) Implementing the plan requires the mobilization of resources, training, organizational culture, reporting systems, and continuous research. (3) The analysis indicates that, despite its good intentions, there are challenges and gaps that need to be overcome.
(Peddle et al., 2025) [27]	Experiences and support of Australian nurses who identify as a second victim: A mixed methods study	Australia	Sequential explanatory mixed methods	(1) To Investigate experiences and key elements of effective support programs that promote recovery in nurses who identify as second victims in Australia.	n=43 nurses/ survey and in-depth interviews.	"Empathy and support from trained peer support colleagues were key elements to recovery of nurses who identified as second victims. The role of culture, and family values and beliefs in support of second victims need further investigation. Support programs need to address, not only

Authors and year of publication	Title	Country	Type of study/ Type of Document	Objective(s)	Data collecting / Sample/ Setting	Main Outcomes
						psychological consequences of a patient safety event on the nurse, but also physical ramifications. Support of supervisors and organisations following a patient safety event may facilitate retention of nurses. The role of education and training in support programs requires further investigation".
(Pimenta, 2021) [50]	*Apoio aos profissionais de saúde: a exposição a incidentes de segurança do doente e a existência de estruturas de suporte* (Support for healthcare professionals: exposure to patient safety incidents and the existence of support structures).	Portugal	Master Thesis	(1) To understand the impact of patient safety incidents on healthcare professionals and to identify the existence of support structures.	Three scientific articles were developed based on two distinct methodological approaches. The first article is a systematic literature review, while the second and third articles are descriptive, cross-sectional, and observational studies.	The investigation proved that the effects of patient safety incidents affecting Portuguese healthcare professionals are like those described by professionals in other countries. The study also revealed the forms of support used by Portuguese healthcare professionals and the type of support they would like to receive from institutions. Conclusion: Patient safety incidents have a negative effect on Portuguese healthcare professionals, making it extremely important to develop support programs for the Second Victim. These programs should support healthcare professionals, thus ensuring their well-being, so that they can continue to provide quality patient care.

Authors and year of publication	Title	Country	Type of study/ Type of Document	Objective(s)	Data collecting / Sample/ Setting	Main Outcomes
(Quadros et al., 2022) [32]	Falls Suffered by Hospitalized Adult Patients: Support to the Nursing Team as the Second Victim	Brazil	Exploratory and Descriptive Study with a Qualitative Approach	(1) To describe the support received by the second victim in falls suffered by hospitalized adult patients from the nursing team's perspective	n= 21 nursing professionals from inpatient units/ semi-structured interviews.	"From the nursing team's perspective, support for the second victim was considered incipient by the hospital institution. On the other, participants highlighted the support received by their families and peers in the work environment. An institutional flow of support for the second victim needs to be formalized to mitigate the repercussions on staff".
(Quillivan et al., 2016) [33]	Patient Safety Culture and the Second Victim Phenomenon: Connecting Culture to Staff Distress in Nurses	USA	Cross-sectional survey study	(1) investigate the effect of patient safety culture on health care provider second victim related distress (2) explore whether patient safety culture affects the degree to which second victims are supported in the aftermath of event involvement.	n=358 nurses at a specialized paediatric hospital, 169 (47.2%)/ Surveys	The results suggest that punitive safety cultures may contribute to self-reported perceptions of second victim–related psychological, physical, and professional dis tress, which could reflect a lack of organizational support. Reducing punitive response to error and encouraging supportive coworkers, supervisors, and institutional interactions may be useful strategies to manage the severity of second victim experiences.
(Research Group on Second and Third Victims , 2015) [53]	Recommendations for Providing an Appropriate Response when patients experience an adverse event with support for	Spain	Guideline	(1) To recommend best practices.	*Not applicable*	Recommendations for providing an appropriate response when patients experience an adverse event with support of healthcare's second and third victims.

Authors and year of publication	Title	Country	Type of study/ Type of Document	Objective(s)	Data collecting / Sample/ Setting	Main Outcomes
	healthcare's second and third victims					
(Sahay et al., 2023) [7]	Nurses and nursing students as second victims: A scoping review.	Australia	Scoping Review	(1) To describe and understand what is known about nurses and nursing students as second victims.	Three databases: CINAHL, Medline, and Proquest for the period between 2010 and 2022. A total of 23 papers underwent thematic analysis.	Nurses and nursing students' well-being and productivity levels can be negatively affected by inadequate team and organizational support. To improve team functioning, appropriate support mechanisms must be implemented to assist nurses who experience significant distress after making errors. Nursing leadership should prioritize improving support programs, assessing workload allocation, and increasing awareness amongst leaders of the potential benefits of providing support to second victims.
(Stone, 2020) [46]	Second Victim Support: Nurses' Perspectives of Organizational Support After an Adverse Event	USA	Qualitative descriptive approach	(1) To describe hospital nurses' experiences with organizational support after an adverse event (AE).	n=12 nurses from ten different Institutions/ One-on-one, semi structured audio-recorded interviews	"Nurses yearn to feel valued and to receive timely support from nurse executives after an AE. To help lessen the suffering of the nurse after an AE, healthcare organizations and nurse executives must support the nurse in the aftermath".
(Strametz et al, 2021) [12]	Prevalence of Second Victims, Risk Factors, and Support Strategies among German Nurses (SeViD-II Survey)	Germany	Nationwide cross-sectional online study	(1) To investigate second victim Syndrome (SVP) in German nurses regarding prevalence, causes, and predisposition compared to	n= 332 nurses/ National Survey (modified SeViD questionnaire including the BFI-10 (personality traits).	"SVP is common among German nurses and comprises other causes and a different course than in physicians. Further research should concentrate on specific prevention strategies, e.g., professional- and workplace-

Authors and year of publication	Title	Country	Type of study/ Type of Document	Objective(s)	Data collecting / Sample/ Setting	Main Outcomes
				a preceding study on German physicians (Second Victims in Deutschland/SeViD-I).		based educational programs". 59% of nurses experience SVP at least once a lifetime.
(Thompson et al., 2022) [4]	Implementation of a Certified Registered Nurse Anaesthetist Second Victim Peer Support Program	England	Quality improvement Project	(1) To decrease second victim distress among certified registered nurse anaesthetists (CRNAs) by implementing a peer support program	n= 8 Certified registered nurse anaesthetists (CRNAs).<br>Pre- and post-implementation second victim<br>distress was assessed using the Second Victim Experience and Support Tool (SVEST).	"The program experienced higher utilization compared to similar launch studies, with eight encounters in the first month. Impact on staff morale is expected to increase; long-term peer support can improve provider well-being and patient outcomes".
(Vanhaecht et al., 2022) [54]	An Evidence and Consensus-Based Definition of Second Victim: A Strategic Topic in Healthcare Quality, Patient Safety, Person-Centeredness and Human Resource Management	Belgium	Guideline	(1) To advance the theoretical conceptualization and to develop a common definition.	Literature search was performed<br>in Medline, EMBASE and CINAHL (October 2010 to November 2020).	"Any health care worker, directly or indirectly involved in an unanticipated adverse patient event, unintentional healthcare error, or patient injury and who becomes victimized in the sense that they are also negatively impacted".

Authors and year of publication	Title	Country	Type of study/ Type of Document	Objective(s)	Data collecting / Sample/ Setting	Main Outcomes
(World Health Organization [WHO], 2021) [55]	Global Patient Safety Action Plan 2021–2030 Towards eliminating avoidable harm in health care	Switzerland	Guideline	(1) To inspire, educate, empower and protect health professionals so that they contribute to the design and delivery of safe care systems	*Not applicable*	Within the objective, strategy 5.5 is dedicated to "designing care settings, environments and practices that provide safe working conditions for all staff". Safe working conditions include improving well-being.
